# The pregnancy and oncology outcome of fertility-sparing management for synchronous primary neoplasm of endometrium and ovary

**DOI:** 10.1186/s13048-023-01316-w

**Published:** 2023-12-08

**Authors:** Qujia Gama, Shuhan Luo, Pengfei Wu, Lulu Wang, Sijia Liu, Hongwei Zhang, Li Sun, Yiqin Wang, Min Yu, Xiaojun Chen, Weiwei Shan, Xuezhen Luo

**Affiliations:** 1 Department of Gynecology, Obstetrics and Gynecology Hospital of Fudan University, 128 Shenyang Road, Yangpu District, Shanghai, 200090 P.R. China; 2 Shanghai Key Laboratory of Female Reproductive Endocrine-Related Diseases, Shanghai, 200090 China; 3 Department of Cervix, Obstetrics and Gynecology Hospital of Fudan University, Shanghai, 200090 China; 4 Department of Ultrasound, Obstetrics and Gynecology Hospital of Fudan University, Shanghai, 200090 China; 5 Department of Pathology, Obstetrics and Gynecology Hospital of Fudan University, Shanghai, 200090 China; 6 Department of Assisted Reproduction, Obstetrics and Gynecology Hospital of Fudan University, Shanghai, 200090 China

**Keywords:** Fertility-sparing treatment, Synchronous endometrial and ovarian neoplasm, Synchronous endometrial and ovarian cancers (SEOC), Endometrial cancer (EC), Ovarian cancer (OC)

## Abstract

**Purpose:**

To investigate the efficacy of fertility-preserving treatment for young women with synchronous primary neoplasm of endometrium and ovary.

**Methods:**

We retrospectively reviewed eight patients with concurrent primary grade 1 presumed stage IA endometrioid endometrial adenocarcinoma (EEA) or endometrial atypical hyperplasia (EAH) and primary stage I ovarian tumors who underwent fertility-sparing treatment in the Obstetrics and Gynecology Hospital of Fudan University between April 2016 and December 2022.

**Results:**

Synchronous endometrial and ovarian cancers (SEOC) accounted for 50% of these eight patients. The median age of patients was 30.5 years (range, 28–34 years). None of them received chemotherapy. The median treatment time was 4 months (range, 3–8 months). 87.5% (7/8) cases achieved complete response (CR), and the median time to CR was 3.8 months (range, 1.5–7.7 months). Among patients who got CR, none of them showed any signs of recurrence. Pregnancies and successful deliveries were achieved in 4 of 5 patients. Till September 2023, the median follow-up period was 50.5 months (range:15.2–85.2 months).

**Conclusion:**

Fertility-sparing treatment is feasible for highly selected patients with synchronous neoplasm of the endometrium and ovary, but strict screening and monitoring are mandatory. Though the results of our limited cases are encouraging, long follow-up and more clinical data are required. Enrolled patients must be fully informed of the risks during conservative treatment.

## Introduction

Synchronous cancers in the female genital tract have been estimated to be 1-2% of all gynecological tumors [1, 2], and synchronous endometrial and ovarian cancers (SEOC) account for about 50-70% of gynecological primary synchronous cancers, 10% of all ovarian cancer (OC) and 5% of all endometrial cancer (EC) [2, 3]. SEOC in young nulliparous women has also been reported. Undoubtedly, the standard treatment for these patients is staging surgery. But this treatment precludes future fertility and may be unacceptable for young women who want to preserve their fertility.

Fertility-sparing management for selected OC, borderline ovarian tumor (BOT), early-stage endometrioid endometrial adenocarcinoma (EEA), and endometrial atypical hyperplasia (EAH) has been described in abundant literature [4–7]. Nevertheless, there is limited literature regarding conservative therapy for young women with SEOC, synchronous EAH and BOT, or synchronous EEA and BOT. To date, only one SEOC case was reported to take conservative treatment and get a spontaneous pregnancy and a normal delivery [8].

Here we reviewed eight cases with synchronous endometrial and ovarian neoplasms, including SEOCs, the concurrence of presumed stage IA EEA and BOT, and the coexistence of EAH and BOT. Through analyzing the oncological and reproductive outcomes of these cases, we aim to share and add knowledge to the conservative treatment of SEOCs, synchronous EEA and BOT, and synchronous precancerous endometrial lesions and BOTs.

## Methods

This is a single-institution retrospective observational study performed at the gynecology department of the Obstetrics and Gynecology Hospital of Fudan University. The medical records of young patients diagnosed with synchronous primary neoplasm of endometrium and ovary, who chose fertility-sparing treatment, were retrospectively reviewed between January 2010 to December 2022 at the Obstetrics and Gynecology Hospital of Fudan University. This study was under review and approved by the ethics committee of the Obstetrics and Gynecology Hospital of Fudan University (ID: 2023-31).

Patients enrolled must meet the following inclusion criteria: the patients (1) must have a strong desire to preserve fertility; (2) were 40 years old or younger; (3) had pathologically assured grade 1 EC or EAH without myometrial invasion, while diagnosed with early-stage well-differentiated EOC or BOT; (4) had no evidence of lymph node involvement or extrauterine lesions (for EC) and ovarian lesions were confined to the ovary, presumed to be stage IA EC and stage I OC based on the International Federation of Gynecology and Obstetrics (FIGO) staging standards released in 2014 [9]; (5) had no liver or kidney dysfunction; (6) had no combined malignancies; (7) had good reliance and (8) had no other contraindications to hormonal therapy and hysteroscopic examination. Regarding pathological diagnosis, two pathologists specialized in gynecologic oncology from the pathology department of the Obstetrics and Gynecology Hospital gave the same diagnosis. In case of discrepancy, a discussion meeting would be held to make a final decision. All enrolled patients were fully informed that conservative therapy is not the standard treatment and requires regular hysteroscopic examination and strict imaging follow-up during the treatment period. All patients signed informed consent and were counseled extensively on the advantages, disadvantages, and risks of surgery and conservative therapies.

Before starting conservative therapy, a comprehensive assessment was performed, including the patient’s basic medical history, metabolic condition, pelvic examination, ultrasound scanning, enhanced pelvic magnetic resonance imaging (MRI) or computed tomography (CT) scanning, enhanced abdominal MRI or CT examination, and hysteroscopic assessment. Fasting blood was drawn to examine liver and renal functions, fasting lipids, fasting blood glucose (FBG), postprandial blood glucose (PBG), fasting insulin (FINS), and hormone levels (including estradiol, progesterone, testosterone, follicle-stimulating hormone (FSH), luteinizing hormone (LH), Anti-Müllerian Hormone (AMH)), and serum tumor biomarkers (CA125, HE4). Homeostasis model assessment-insulin resistance (HOMA-IR) was calculated as FBG (mmol/L) × FINS (µU/mL)/22.5, and insulin resistant (IR) and metabolic syndrome (MS) were assessed [10, 11], and IR was diagnosed when the HOMA-IR was over 2.95 as indicated in previous research [11]. Lynch screening with immunohistology staining was performed in each patient, and each case accepted genetic counseling.

Concerning medication, continuous oral megestrol acetate (MA) 160 mg daily or combined with a levonorgestrel-releasing intrauterine device (LNG-IUD) was used. Metformin was also used in some cases because of its potential benefits [12, 13]. The combination of intramuscular injection of 3.75 mg gonadotropin-releasing hormone agonist (GnRHa) every 4 weeks plus oral letrozole 2.5 mg daily was also adopted. The regular hysteroscopic examination was performed every 12–16 weeks to assess endometrium and regular imaging follow-up was also executed to monitor pelvic and abdominal fields to exclude further progression during conservative management. Towards ovarian lesions, unilateral adnexectomy or cystectomy was performed for unilateral EOC or BOT, and unilateral adnexectomy plus contralateral cystectomy was performed for one case with bilateral BOTs. None of them received chemotherapy. For Case3 with stage IA ovarian cancer having local dedifferentiated carcinoma, chemotherapy was strongly recommended, but the patient rejected it.

The treatment response of endometrial lesions was assessed every 3–4 months and was categorized as follows. No evidence of any cancerous lesions and endometrial hyperplasia was classified as complete response (CR). For primary EC patients, being present with EAH or benign hyperplasia without cancerous lesions was considered as partial response (PR). For primary EAH patients, endometrial lesions recessed into benign hyperplasia, simple or complex hyperplasia, was classified into PR. No response (NR) referred to the continued presence of primary endometrial lesions without pathological improvement. Progressive disease (PD) was annotated as progressed disease, such as EAH developed into EC, or evidence of myometrial invasion occurred in primary EC patients.

If CR was not achieved within 10 months or NR persisted over 6 months, surgery would be strongly suggested. For patients with CR, assisted reproductive technology (ART) was suggested for fertility. Levonorgestrel-intrauterine system (LNG-IUS) or oral contraceptives or dydrogesterone was used during ART to protect endometrium. Maintenance treatment, LNG-IUS or oral contraceptives was introduced for patients without family planning to prevent recurrence. Intense long-term follow-up was emphasized for all CR patients. Surveillance included periodic interviews of any abnormal symptoms, physical examinations, serum tumor biomarkers, and transvaginal ultrasonography. Further imaging, like enhanced CT scanning or enhanced MRI, would be performed if necessary. Besides regular endometrial biopsy would be carried out every 6 months and hysteroscopic examination and laparoscopic examination would be performed if necessary.

For reproductive outcomes, the pregnancy rate was defined as the percentage of women who succeeded in achieving pregnancy among CR patients attempting to conceive. The live birth rate was calculated through live births divided by successful pregnancies in CR patients.

## Results

### Basic clinical characteristics of enrolled patients

Eight patients were enrolled in this study between 2016 and 2022 (As shown in Table 1). The median age of the eight patients was 30.75 years (range, 28–34 years). Among them, four (50%) patients had SEOC, two (25%) patients were diagnosed as EAH with synchronous BOTs, and the other two (25%) patients had concurrent endometrial cancer and BOTs. Half of the patients presented with abnormal uterine bleeding (AUB) plus adnexal mass, two other cases initially complained of abdominal pain and adnexal mass, and the other two cases primarily presented with AUB. One patient (12.5%) had metabolic syndrome and presented with hyperlipemia, type 2 diabetes, and insulin resistance. Though 62.5% (5/8) of the patients had a family history of cancers, lynch syndrome screening was negative for the whole eight patients, while four of them took germline genetic screening and had no significant findings. Three cases (37.5%) showed increased levels of tumor biomarkers (CA125 and HE4) at baseline and normal values after treatment. Detailed information on the eight patients’ treatment regimens, therapeutic responses, and oncological and reproductive outcomes are shown in Table 2.

**Table 1:** Demography and clinical characteristics of eight patients with synchronous primary endometrial and ovarian neoplasm

NO	Age	Complaint	Hyperlipemia	Type2 Diabetes	IR	MS	Family history of cancers	Lynch screening	Germline mutation testing	CA125 (U/mL)	HE4 (pmol/L)	Pathology of endometrium	Immunohistochemistry of endometrial lesions	Pathology of ovarian tumors	Originated from endometrioid cysts	FIGO staging of ovarian tumor
Case1	32	AUB, adnexal mass	NO	NO	NO	NO	YES	Negative	No data	21	94.4	EEA, G1	ER(+ ,60%),PR(+ ,10%),Ki-67(+ ,1%),P53(+ , wild type)	Endometrioid carcinoma	YES	IA
Case2	28	AUB	NO	NO	NO	NO	NO	Negative	No data	17.7	38.5	EEA, G1	ER(+ ,80%),PR(+ ,10%),Ki-67(+ ,8%),P53(+ ,wild type)	Endometrioid carcinoma	NO	IA
Case3	34	AUB, adnexal mass	YES	YES	YES	YES	YES	Negative	Negative	65	147	EEA, G1	ER(+ ,80%), PR(+ ,80%), Ki-67(+ ,10%),P53(+ ,wild type)	Endometrioid carcinoma with local dedifferentiated carcinoma	YES	IA
Case4	34	abdominal pain and adnexal mass	NO	NO	NO	NO	NO	Negative	Negative	62	195	EEA, G1	ER(+ ,80%),PR(+ , 80%),Ki-67(+ ,40%),P53(+ ,wild type)	Endometrioid carcinoma	NO	IC1
Case5	29	AUB, adnexal mass	NO	NO	NO	NO	YES	Negative	No data	24	35.7	EEA, G1	ER(+ ,70%), PR(+ ,80%), Ki-67(+ ,20%),P53(+ ,wild type)	Endometrioid borderline tumor	YES	IC1
Case6	30	abdominal pain and adnexal mass	NO	NO	NO	NO	NO	Negative	Negative	15.97	41.7	EEA, G1	ER( +),PR( +),Ki67(+ ,5%),p53(+ ,wild type)	Endometrioid borderline cystadenoma	YES	IA
Case7	28	AUB	NO	NO	NO	NO	YES	Negative	Negative	23.6	35.7	EAH	ER(+ ,95%),PR(+ ,60%),Ki-67(15% + ,P53(+ ,wild type)	Serous borderline tumor	NO	IC
Case8	31	AUB, adnexal mass	NO	NO	NO	NO	YES	Negative	No data	18	41.5	EAH	ER(+ ,90%),PR(+ ,90%),Ki67(+ ,30%),P53(+ ,wild type)	Endometrioid borderline cystadenoma	YES	I

*Abbreviations: AUB* Abnormal uterine bleeding, *IR* Insulin resistance, *MS* Metabolic syndrome, *CA125* Carbohydrate antigen125(the normal values for CA125 is < 35.00U/ml), *HE4* Human epididymis protein4(the normal values for HE4 is < 60.5 pmol/L in females younger than 40;) *EAA* Endometrioid endometrial adenocarcinoma, *EAH* Endometrial atypical hyperplasia, *ER* Estrogen receptor, *PR* Progesterone receptor

**Table 2:** Oncologic and fertility outcomes of the eight patients

NO	Lesions	Treatment for endometrial lesions(months)	Treatment response of endometrial lesions	Time to CR (months)	Maintenance treatment after CR	Treatment for ovarian tumors	Chemotherapy	Pregnancy	Interval time between CR and the initial pregnancy(months)	Live birth	Status	Follow-up (months)	Recurrence
Case1	SEOC	MA + LNG-IUD + MET(4)	NR (hysterectomy)	——	——	Right adnexectomy	No	——	——	——	Hysterectomy	NED (71)	No
Case2	SEOC	MA(8)	CR	7.7	Diane-35	Left cystectomy	No	1(IVF-ET)	10.6	1(38W CS)	Total hysterectomy + bilateral salpingo-oophorectomy	NED (55.7)	No
Case3	SEOC	MA + LNG-IUD + MET(8)	CR	6.6	Diane-35 + MET	Right adnexectomy	No	0(IVF-ET)	——	——	Diane + MET	NED (47.2)	No
Case4	SEOC	GnRH-a + letrozole(4)	CR	3.3	Diane-35 + MET	Left adnexectomy + pelvic lymph dissection + aortic lymph biopsy + peritoneal biopsy + omentum excision	No	0(Unmarried)	——	——	Diane + MET	NED (15.2)	No
Case5	EEA + BOT	MA + MET(4)	CR	1.8	LNG-IUD	Left adnexectomy after cystectomy	No	1(IVF-ET)	18.3	1(32W CS)	Total hysterectomy + right salpingectomy	NED (29.4)	No
Case6	EEA + BOT	GnRH-a + letrozole(4)	CR	3.8	Diane-35 + MET	Right cystectomy	No	1(IVF-ET)	12.5	1(38W CS)	lactational period	NED (24.4)	No
Case7	EAH + BOT	MA(6)	CR	3.8	Diane-35 + MET	Left cystectomy + right adnexectomy	No	0(Family planning)	——	——	LNG-IUD	NED (85.2)	No
Case8	EAH + BOT	MA + MET(3)	CR	1.5	Diane-35 + MET	Left adnexectomy	No	1(IVF-ET)	12.8	1(34W CS)	LNG-IUD	NED (53.9)	No

*Abbreviations: MA* Megestrol acetate, *LNG-IUD* Levonorgestrel-releasing intrauterine device, *MET* Metformin, *GnRHa* Gonadotropin-releasing hormone agonist, *NR* No response, *CR* Complete response, *IVF-ET* In-vitro fertility embryo transplantation, *CS* Caesarean section, *NED* No evidence of disease

### Characteristics of ovarian and endometrial lesions

As shown in Table 1, the four SEOC patients showed endometrioid carcinoma in both the endometrium and ovary, with one case (Case3) having local dedifferentiated carcinoma under a high-resolution vision. Among the four SEOCs, three had unilateral adnexectomy, and one (Case2) had intact tumor resection without membrane abruption during the operation. Intra-operatively fasting-frozen pathology indicated as a BOT and post-operatively the formalin-imbedded pathology showed BOT with local ovarian endometrioid carcinoma, secondary adnexectomy was not performed.

Four BOT cases presented with three endometrioid borderline cystadenomas and one serous borderline cystadenoma, while two cases coexisted with endometrial cancer and the other two cases had endometrial atypical hyperplasia. Two patients with BOTs had unilateral adnexectomy, and one had cystectomy, for Case7 with bilateral BOTs, she had right adnexectomy plus left cystectomy.

Five of the eight ovarian lesions originated from endometriotic cysts. All endometrial lesions harbored wide-type P53 expression and positive estrogen receptor (ER) and progesterone receptor (PR) expression, which may be attributed to the favorable treatment response to some extent in our study.

### Fertility outcomes

As indicated in Table 2, seven patients (87.5%) achieved CR, five (71.4%) cases underwent in vitro fertilization and embryo transfer (IVF-ET), and the other two cases had no family planning recently and LNG-IUS was inserted in the uterine for prevention. Among the five IVF-ET patients, 80% (4/5) got pregnant had cesarean section, and had four live births. Among the four deliveries, Case2 had a planned cesarean at 38.2 weeks pregnant, followed by a staging surgery immediately, and Case5 and Case8 had emergent cesareans due to severe eclampsia and placental abruption respectively, and Case6 had a planned cesarean at 38.4 weeks pregnant this July and in the lactational period. The interval time between CR and the initial pregnancy of Case 2, Case 5, Case 6, and Case 8 was 10.6, 18.3, 12.5, and 12.8 months, respectively. Case 3 had bad reliance after CR and rejected further medical treatment for diabetes and obesity. Her ovarian function was poor, and no good eggs could be drawn, and she failed in vitro fertilization twice unexpectedly. The staging surgery was strongly suggested, but the patient rejected it.

### Oncologic outcomes

As depicted in Table 2, among the eight patients, seven (87.5%) patients achieved CR with a median treatment time of 4 months (range, 3–8 months), and the median time to CR was 3.8 months (range, 1.5–7.7 months). Till September 2023, three patients chose operation. Case 1 gave up fertility-sparing treatment and chose hysterectomy plus left salpingectomy after taking intermittent 4-month MA treatment, and the post-operative histopathological result showed EC with superficial myometrial invasion. Case 2 had staging surgery immediately after cesarean section, and no residual lesions were found pathologically. Case 5 chose hysterectomy plus right salpingectomy three months after cesarean section. Of the left five cases, Case6 is in a lactational period now and the other four cases are under maintenance treatment, two (Case7, Case8) chose LNG-IUD and the other two (Case3, Case4) are taking Diane-35 plus metformin for prevention. Till September 2023, the median follow-up time is 50.5 months (range, 15.2–85.2 months). No recurrence was found. No disease-related severe events nor medication-related adverse effects were found in this study.

## Discussion

Though the incidence of synchronous endometrial and ovarian cancers is low, it shouldn’t be omitted as the number of young nulliparous endometrial cancer and ovarian cancer patients is increasing [14, 15]. Our study is the first to report eight patients with concurrent endometrial and ovarian lesions who underwent fertility-sparing management, including 4 SEOCs, 2 BOTs concurrent with EC, and 2BOTs combined with EAH. Looking through the current literature, four SEOC cases were reported to take fertility-sparing therapy [8, 16–18], but two of the four cases terminated fertility preservation after confirming SEOC, and one case chose hysterectomy with ovarian conservation after 7-month progesterone therapy in the hope of gestational surrogacy, and the case took staging surgery after oocyte retrieval, and only one patient insisted conservative therapy and succeeded and got spontaneous pregnant after operation and six cycles of chemotherapy. In our study, the complete remission rate was 87.5% (7/8) while only one got NR terminated conservative treatment halfway. The pregnancy rate was 80% (4/5), with four live births. Though successful cases and related experience are limited, fertility-sparing treatment for SEOC patients and concurrent BOTs and EC and EAH patients should be explored further.

### Feasibility

Nowadays no standard guidelines in this field, but fertility conservative treatment for SEOCs or concurrent precancerous endometrial and ovarian lesions seems not impossible, according to our study and the published cases. It also seems feasible and explorable from other perspectives. Firstly, limited available data or experience directly supports conservative treatment in SEOCs or concurrent endometrial and ovarian malignancies, but highly selected patients may deserve the try. Tons of evidence has shown that elected patients with early presumed IA EC and EAH can undertake fertility-sparing treatment and get good outcomes [4], which can also be seen in BOTs [19] and some OCs [5, 20] within certain pathological profiles. Secondly, SEOC patients usually have earlier tumor stages, lower pathological grades, and better prognoses, compared to single EC and OC patients [21]. According to previous studies, the estimated 5-year overall survival (OS) of SEOC patients is 79.7-85.9%and 10-year OS is about 72.5-85.6% [3, 22, 23], which is similar to stage I EC without synchronous OC. Though the above data is from patients with staging surgery, its favorable prognosis provides the possibility of fertility preservation for highly selected SEOC patients. The median age of SEOC patients is about 10 years younger than patients with single EC or OC, and about 30% of them have not yet given birth [22]. As stated in 2021 ESGO guidelines [24], indolent behavior of SEOC with low-grade endometrioid carcinoma supports conservative management when the following criteria are met: (a) both tumors are low grade; (b) < 50% myometrial invasion; (c) no involvement of any other site; (d) absence of extensive LVSI at any location. Thirdly, with the development of molecular diagnosis, genetic screening, imaging techniques, and laparoscopy, as well as hysteroscopy, pathologists, and clinicians have more confidence in differentiating synchronous cancers or metastasis, which is of pivotal significance in fertility-preservation management. Based on the above evidence, fertility-sparing treatment is worth trying in this population, though it is challenging.

### Risks

Risks and challenges coexist. Differentiation between “metastatic” and “synchronous” is the primary determinant before moving towards conservative management. Adnexal involvement by endometrial cancer or uterine involvement by OC is currently an indicator affecting FIGO staging and has an impact on patients’ overall survival rates. Patients with synchronous involvement of the endometrium and ovary by low-grade carcinoma had a favorable outcome [22]. In our study, all four SEOC patients had the synchronous presentation of low-grade endometrioid endometrial and ovarian carcinomas, and one case (Case3) had local dedifferentiated carcinoma in OC. By the way, five cases of ovarian lesions originated from ovarian endometriotic cysts in our study, which also helps pathologists and clinicians judge between “metastatic” and “synchronous”. Though several criteria [25, 26] have been suggested in the past to help distinguish between metastatic tumors and synchronous primary tumors, it is not easy to apply.

And recent studies [27, 28] have indicated that there is a clonal relationship between EC and OC in SEOC patients, the theory of “restricted metastatic potential” or “restricted dissemination” has been proposed by Anglesio [29], and such patients do not need adjuvant therapies [24]. This theory [29] regards that the tumor is isolated from the primary site, and the disseminated tumor is confined to the new site due to the effect of the microenvironment at the new site. The tumor cells detach from a primary site without apoptosis, spread, and recolonize at certain areas under a strict microenvironment without the capacity for further dissemination [27, 29]. Nowadays traditional histological diagnosis is still the most powerful and effective method in the clinic, molecular analysis may also help, and we need to explore more methods in differentiating “metastatic” and “synchronous”.

Besides careful pathological examination, other aspects should also be fully assessed, including patients’ desire for fertility, ovarian function, metabolic conditions, and reliance. No standard treatment is advised. High dosages of high-efficacy progesterone, LNG-IUD, and GnRha were used in our study, but which one is the best choice? We are not sure yet. More studies are needed. The regular hysteroscopic examination is used for endometrium evaluation while imaging and serum biomarkers are used for monitoring potential extra-uterine metastasis. Micro-metastasis or potential progression may be hard to detect at an early stage. We suggest experienced experts and a multiple-disciplinary team participate in treatment, monitoring, and long-term follow-up during the whole period of conservative management, and extensive follow-up should be extended into the periods of assisted reproductive treatment, stages of pregnancy, and post-reproduction. Lots of related problems are needed to be addressed in the future.

### Oncological and reproductive outcomes and ART challenge

In our study, the CR rate (87.5%), the pregnancy rate (80%), and the live birth rate (80%) are promising, and the oncologic and reproductive outcomes are exciting. But long-term outcomes are still unclear, and we will continue to follow up on each case. Besides treating endometrial and ovarian lesions, how to choose the proper ART after CR is also a big challenge, especially during the process of ovarian stimulation. Different studies on the associations between fertility drug use and potential OC risk and endometrial cancer risk are conflicting [30–32]. Though most studies showed no meaningful increased risk of invasive OC or uterine cancer related to fertility drug use, those researches mainly focused on the infertility population not affected by genital cancers. There is no robust research focused on fertility drug use in females affected by EEA OC BOTs or SEOC. Limited observations or cohort data are available, so we still need to be cautious when choosing ovulation-stimulating agents for those females. Ovulation stimulation protocols include conventional short agonist regimen, long agonist regimen, antagonist regimen, mild stimulation protocol (usually clomiphene citrate (CC) or letrozole (LE) combined with gonadotropins (Gn)), and progestin-primed ovarian stimulation (PPOS). We prefer PPOS and mild stimulation protocol to other protocols in the hope of minimizing the risk of recurrence. However it’s still not clear which ovarian stimulation protocol is the most effective and safe for those patients, and more in-depth and larger sample studies are needed.

### Limitations

This is a single-center retrospective study. Cases are limited and more cases should be collected and studied to add knowledge to this field. The available evidence is not robust, and more large-scale studies are warranted to induce experience and guidelines in managing young women with early-stage SEOC or coexistence of ovarian and endometrial neoplasms who want to preserve fertility. And the follow-up time is not long enough which may limit our assessment of recurrence and long-term prognosis, and we will continue to follow up.

## Conclusion

In conclusion, fertility-sparing treatment towards early-stage SEOC or EEA/BOT or EAH/BOT patients is worth trying and further exploration. Close monitoring and management are mandatory during and after fertility-sparing treatment for those highly selected patients. More high-quality clinical studies are urgently needed.

## Data Availability

The datasets generated for this study are available on request to the corresponding author.
